# Seasonality in mortality and its relationship to temperature among the older population in Hanoi, Vietnam

**DOI:** 10.3402/gha.v7.23115

**Published:** 2014-12-08

**Authors:** Le Thi Thanh Xuan, Thaddaeus Egondi, Le Tran Ngoan, Do Thi Thanh Toan, Le Thi Huong

**Affiliations:** 1 Department of Environmental Health, Institute for Preventive Medicine and Public Health, Hanoi Medical University, Hanoi, Vietnam; 2 African Population and Health Research Center, Nairobi, Kenya; 3 Epidemiology and Global Health, Department of Public Health and Clinical Medicine, Umeå University, Umeå, Sweden; 4 Department of Occupational Health, Institute for Preventive Medicine and Public Health, Hanoi Medical University, Hanoi, Vietnam; 5 Department of Biostatistics and Medical Informatics, Institute for Preventive Medicine and Public Health, Hanoi Medical University, Hanoi, Vietnam; 6 Department of Nutrition and Food Safety, Institute for Preventive Medicine and Public Health, Hanoi Medical University, Hanoi, Vietnam

**Keywords:** temperature, mortality, older, Vietnam

## Abstract

**Background:**

Several studies have established a relationship between temperature and mortality. In particular, older populations have been shown to be vulnerable to temperature effects. However, little information exists on the temperature–mortality relationship in Vietnam.

**Objectives:**

This article aims to examine the monthly temperature–mortality relationship among older people in Hanoi, Vietnam, over the period between 2005 and 2010, and estimate seasonal patterns in mortality.

**Methods:**

We employed Generalized Additive Models, including smooth functions, to model the temperature–mortality relationships. A quasi-Poisson distribution was used to model overdispersion of death counts. Temporal trends, seasonality, and population size were adjusted for while estimating changes in monthly mortality over the study period. A cold month was defined as a month with a mean temperature below 19°C.

**Results:**

This study found that the high peak of mortality coincided with low temperatures in the month of February 2008, during which the mean temperature was the lowest in the whole study period. There was a significant relationship between mean monthly temperature and mortality among the older people (*p*<0.01). Overall, there was a significant decrease in the number of deaths in the year 2009 during the study period. There was a 21% increase in the number of deaths during the cold season compared to the warm season. The increase in mortality during the cold period was higher among females compared to males (female: IRR [incidence relative risk] =1.23; male: IRR=1.18).

**Conclusions:**

Cold temperatures substantially increased mortality among the older population in Hanoi, Vietnam, and there were gender differences. Necessary preventive measures are required to mitigate temperature effects with greater attention to vulnerable groups.

Globally, the older population is growing rapidly in developing countries, and this has increased attention toward understanding health risks among older people. Likewise, the older population in low- or middle-income countries is suspected to be at higher risk of death due to weather variations (1–10). The reason might be partly attributable to developing countries’ limited capacity to address health problems in general but also, more particularly, to a lack of health interventions targeting older populations in these countries. Older people also have a reduced ability to maintain a normal temperature of 37°C (11–13). Furthermore, evidence indicates that factors such as poor living conditions, lack of access to proper medical care systems, and lack of family and/or social support have been shown to be modifiers of temperature-related effects among older people (14). These factors are worse in most developing countries, and, therefore, the effect of temperature among older people is expected to be greater. However, little evidence exists on temperature-related impacts among older populations in low- and middle-income countries.

Recently, there has been a growing body of literature on the temperature–mortality relationship among older people in both developed (13, 15–23) and developing countries (2, 4, 5, 8, 9, 24–26). Most previous studies have investigated the association between temperature and mortality, and found significant association (27). Different temperature indicators have been used in previous studies to assess temperature-related mortality. The indicators considered in previous studies included mean temperature (2–4, 10, 25, 26, 28), diurnal temperature (29, 30), and ambient (22, 31) or apparent temperatures (7, 32, 33). All of these indicators are found to produce similar results on temperature-related mortality (10). The temperature–mortality relationship has been found to show U, V, or J shapes, with a U-shaped pattern being a common shape (10, 24). The observed shapes show that, given a particular temperature threshold, increases and decreases in temperature are associated with increases in mortality (2–4, 15–17, 20, 21, 23–25, 34), but the magnitude of heat-related effects seemed to be larger than that of cold effects within a global context (9). Similar observations have been made among older populations (28, 35). Establishing a temperature–mortality relationship is critical for weather-based early warnings and the identification of susceptible groups. In addition, knowledge on temperature effects would show the importance of mitigation and adaptation strategies aimed at reducing the temperature-related effects.

Numerous studies have also investigated seasonal mortality patterns and cold-related excess mortality in the populations living in temperate climate regions (36, 37). So far, there are few such studies from warmer or subtropical areas. Excess mortality during cold temperatures may be a result of such factors as housing conditions, health status and demography, seasonality in infectious disease and indoor crowding, and fuel type (37–40). Studies from Europe show less winter excess mortality in Scandinavian countries (around 10%) compared to Southern European countries (36, 37, 39). The observed difference in winter-related mortality is partly attributed to the adaptation of housing to cold outdoor conditions, which is much more developed in Northern Europe compared to Southern Europe.

Vietnam is one of the countries that experience a tropical and humid climate. Over the last five decades, the temperature has increased by 0.2°C (41). The average temperature is projected to increase by 2.3°C in 2100 in Vietnam (baseline period: 1980–1999), although the changes vary across the country (42, 43). A recent study found that the older population is one of the most vulnerable groups affected by temperature changes in urban areas (44). However, so far, little information exists on the association between mortality and temperature among older populations in Vietnam. The current article aims to examine the seasonality in mortality and to quantify temperature-related mortality among the older population in Hanoi, Vietnam, during the period between 2005 and 2010.

## Methods

### Study location

Hanoi is the capital of Vietnam; it is situated in the north and located at latitude 16°0′ N. It has a population of about 6.5 million people, with 12% aged 60 years and older (45). Hanoi experiences a climate in which summers are hot and humid, and winters are relatively cool and dry (46). The summer period is from May to September, during which the highest amount of rainfall is experienced (1,682 mm rainfall). The winter months from December to March are relatively dry, while light rains are experienced during spring (from 18.6 mm to 23.4 mm). The minimum temperature during winter reaches about 6–7°C with no chilly wind, while summer can get to a maximum of 38–40°C.

### Data collection

We obtained daily weather data from Lang Station on daily mean temperature, minimum and maximum temperature, relative humidity, and rainfall. This station is located at the urban area of Hanoi city. Mortality data were obtained from the Vietnam health system's commune-level health stations. The Vietnam health system consists of four administrative levels: central, provincial, district, and commune levels. There is a policy that every death in Vietnam should be registered at commune health centers. We collected aggregated individual data (for sex and age group) for all 69,690 registered deaths between 1 January 2005 and 31 December 2010 from all 27 commune health centers in Hanoi city. The number of deaths of people aged 60+ was 47,172 cases, representing 68% of the total deaths. The data collected included month of death, sex, age, and location of the diseased person. Therefore, the analysis was conducted on aggregated mortality data at a monthly time scale.

### Data analysis

Summary measures were generated for both weather and death counts (all deaths and deaths among older people). Spearman correlation analyses were conducted using STATA software (47) to assess the bivariate association between the counts of death among older people and temperature measures (minimum (min), maximum (max), and mean), as well as other weather indicators (rainfall and relative humidity). The daily mean temperature was used as a temperature indicator to assess the relationship between weather and mortality. The mean temperature indicator has been shown to produce similar effect estimates as compared to other temperature indicators (min, max, and range) (10).

Counts of monthly deaths and mean temperature were modeled using quasi-Poisson regression to account for overdispersion. The use of quasi-Poisson does not have an effect on the coefficient estimates of the model but adjusts standard errors for overdispersion. The temperature–mortality relationship was examined through Generalized Additive Models (GAMs) with the use of smooth functions. The model included nonlinear effects of weather variables, trend, and season. The model equation for the GAM is described here (48). The Akaike Information Criterion (AIC) was used to assess different models with lags up to three months, while smoothness parameters were determined using graphical visualization. The initial assessment did not show any delay effect of temperature, and therefore the final model did not include lag terms. Lack of lag effect could be because of the aggregation of mortality data at a monthly scale.

$$Y_{t}\sim Poisson\left(\mu_{t}\right)$$

$$\text{log}\left(\mu_{\text{t}}\right)=\alpha +s\left(time,df\right)+s\left(month,df\right)+s\left(tmean,df\right)$$

where *t* refers to the month of the observation; (*Y*_*t*_) denotes the observed monthly death counts during month *t*; *s(.)* denotes a smooth function; *df* represents degrees of freedom, or the smoothing parameter; *tmean* is the mean monthly temperature; and *time* and *month* represent both trend and seasonal terms, respectively. The GAM model was fitted using R statistical software (R Foundation for Statistical Computing, version 3.0).

To quantify the cold-season-related mortality among older people, a dummy variable was created to represent cold and warm seasons. At the initial inspection of the temperature seasonality, the cold season was identified to be during the months of December, January, February, and March with an average temperature of 18.9°C. Quasi-Poisson regression was used to estimate the relative risk related to cold weather using a calendar year as a factor variable. The interaction between season variable and year was checked to examine whether the season effect differed across the years. The interaction term was dropped from the model because it was insignificant, and the final model was:

$$\text{log}\left(\mu_{t}\right)=\beta_{0}+\sum_{i=1}^{5}\beta_{i}Year_{i}+\beta_{6}Season$$

where the year 2005 was taken as the baseline and the season is ‘1’ for cold months. Therefore, the exponential of *β*_6_ gives the relative risk of death among older people during the cold season compared to the warmer season. The model was also fitted for males and females separately. The total population of older people was used as an offset in all quasi-Poisson models. For all statistical tests, two-tailed tests were considered statistically significant with a *p*-value less than 0.05. All data manipulation was done in STATA, and statistical analyses were performed using the ‘mgcv’ functions of R packages.

## Results

Summary statistics for monthly weather conditions (temperature, humidity, and rainfall) and death counts are presented in Table 1. The monthly average estimates for mean, minimum, and maximum temperatures were 24.5°C, 22.0°C, and 28.4°C, respectively. Average relative humidity was 78% for the study period. The average mortality count for all-cause deaths among older people was 662 deaths (346 males and 316 females).

**Table 1:** Summary statistics for monthly weather and number of deaths in Hanoi, Vietnam, 2005–2010

		Percentiles				
		
	Mean (SD)	Min	P_25_	Median	P_75_	Max
Weather measures						
Mean monthly temperature (°C)	24.5 (4.7)	13.8	20.9	25.5	28.7	30.9
Min monthly temperature (°C)	22.0 (4.4)	12.1	18.6	23.1	26.1	27.8
Max monthly temperature (°C)	28.4 (5.1)	16.3	24.1	29.5	32.9	35.5
Rain fall (mm)	112.2 (132.3)	0.4	8.1	40.1	191.7	550.5
Relative humidity (%)	78.1 (44.8)	66.5	75.4	78.5	81	87.6
Wind speed	1.5 (0.2)	0.9	1.3	1.4	1.7	1.9
Monthly all-cause deaths						
Male	564.6 (104.5)	15	514	553	614	903
Female	388.3 (84.2)	9	342	368	438	674
Total	953.0 (185.3)	24	842	927	1,024	1,577
Monthly deaths among older people						
Male	346.9 (58.5)	240	311.5	331	384.5	606
Female	316.7 (61.9)	217	278.5	299	347.5	585
Total	666.1 (121.9)	497	580.5	649	734.5	1,191

Three temperature measures were strongly correlated with each other with a correlation coefficient of between 0.98 and 0.99, but they were not strongly correlated with humidity or rainfall. Among all weather variables, the correlations showed that the three temperature indicators were negatively correlated with mortality with almost similar correlation coefficient estimates. The coefficients ranged from −0.33 to −0.46 with *p*-values less than 0.01 (Table 2).

**Table 2:** Spearman correlation coefficients between mortality and weather variables

	All-cause death: ages 60+	All-cause death: males ages 60+	All-cause death: females ages 60+
T_mean_	−0.42	−0.35	−0.45
T_min_	−0.39	−0.33	−0.43
T_max_	−0.42	−0.36	−0.46
Humidity	0.11	0.09	0.11
Rainfall	−0.06	−0.04	−0.08

**Fig. 1:**
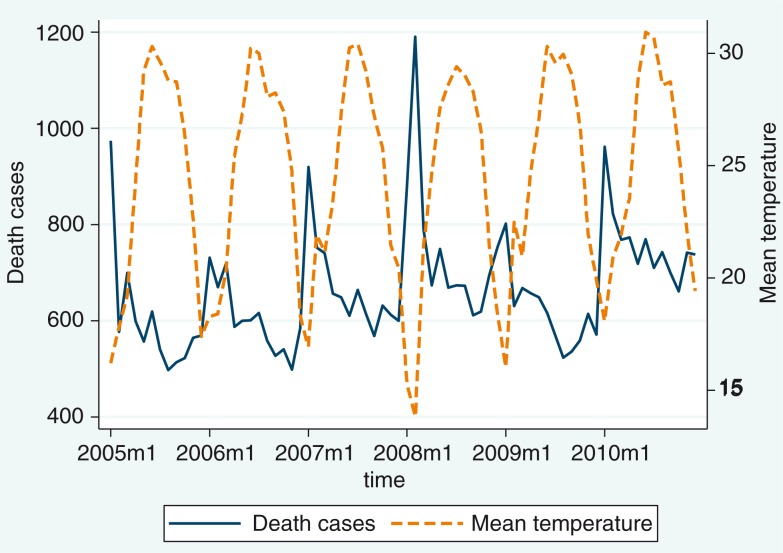
Distribution of monthly reported death cases and mean temperature for the period 2005–2010.

Monthly seasonal variation in the death counts among ages 60+ and in temperature is illustrated in Fig. 1. The plot shows that the high peak of mortality coincided with low temperatures in the month of February 2008, during which the mean temperature was the lowest in the whole study period.

**Fig. 2:**
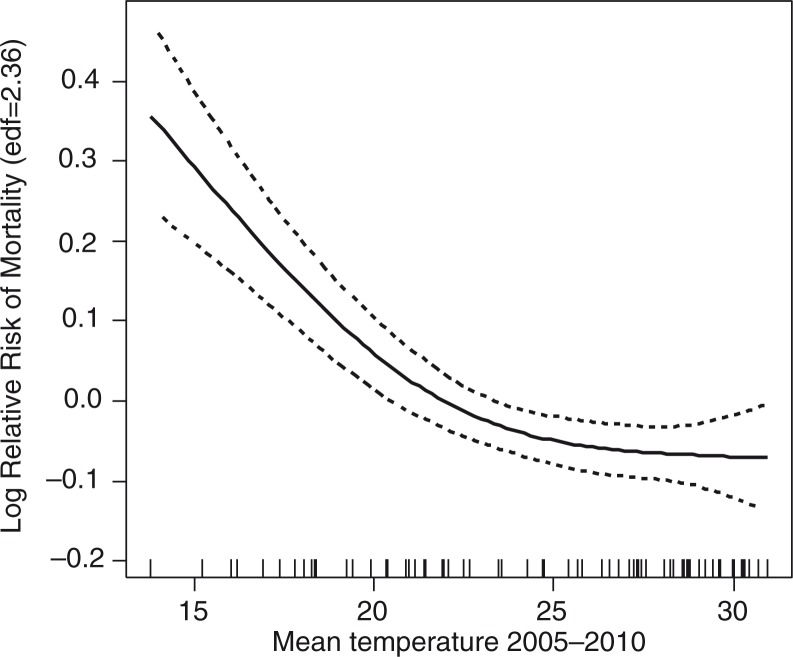
The relationship between monthly mean temperature and all-causes mortality among older people in Hanoi, Vietnam, for the period 2005–2010, adjusted for time trend and seasonality.

The results from the GAM model (Fig. 2) show that there is a significant relationship between mean monthly temperature and mortality among older people (*p*<0.01). The model also shows that there were significant seasonal patterns in the older population's mortality, but no trend in mortality was discernible in this age group. Figure 2 illustrates the nonlinear relationship between temperature and risk of death among older people at the monthly time scale. The curve shows cubic splines of temperature with 2.36 degrees of freedom from the GAM model after controlling for time trend (edf=1.60) and seasonality (month of the year; edf=1.94). The observed relationship implies that a decrease in temperature is associated with an increase in mortality risk.

The results for quantifying the risk related to the cold season are presented in Table 3. We considered the interaction between the period and season, but it was not significant. Therefore, the result for a model without the interaction was presented. Compared to the year 2005, the death cases in 2009 decreased significantly (IRR [incidence relative risk]=0.84, 95% CI=0.77–0.92, *p*<0.05), whereas other years were not significantly different. The mortality risk was 21% higher among the older population during the cold season compared to the warm season. This estimate corresponds to the winter excess mortality. The risk was higher among female older people compared to male ones (male: IRR=1.18; female: IRR=1.23).

**Table 3:** The incidence relative risks (IRRs) between period, season, and death cases in Hanoi, Vietnam, 2005–2010

	Total death cases			Male death cases			Female death cases		
	
	IRR	*p*	95% CI	IRR	*p*	95% CI	IRR	*p*	95% CI
Period (reference: 2005)									
2006	0.95	0.205	0.87–1.03	0.97	0.503	0.89–1.05	0.96	0.414	0.87–1.06
2007	0.96	0.400	0.88–1.05	0.99	0.783	0.91–1.07	0.98	0.714	0.89–1.08
2008	1.03	0.525	0.94–1.12	1.04	0.351	0.96–1.12	1.07	0.165	0.97–1.18
2009	**0.84**	**0.000**	**0.77–0.92**	**0.86**	**0.000**	**0.79–0.93**	**0.87**	**0.003**	**0.79–0.95**
2010	1.01	0.769	0.93–1.10	1.05	0.252	0.97–1.13	1.02	0.627	0.93–1.13
Cold season (reference: warm season)	**1.21**	**0.000**	**1.15–1.28**	**1.18**	**0.000**	**1.12–1.23**	**1.23**	**0.000**	**1.16–1.29**

Bold values denotes for significant difference with *p*<0.001.

## Discussion

This study contributes to an understanding of the temperature–mortality relationship in developing countries. We observed the seasonal effect on mortality among the older population in the capital of Vietnam. The study also found that older people are more susceptible to the effects of cold weather and that females are at greater risk. The observed excess mortality during cold weather in the study area of Vietnam was higher than that observed in Scandinavian countries but lower than that in some Southern European countries (36). However, we acknowledge the difference in time scale between our study and the previous studies, which may contribute to the size of the effect estimate.

In 2009, there was a significant drop in the number of deaths of older people as compared to other years during the study period. This could be due to excess deaths that were observed during the cold period in 2008. In fact, there were 8,935 death cases among the older population reported in 2008, and the cause of high mortality was circulatory diseases at highest level (accounted 26.72%). Some studies have investigated a similar mortality displacement effect due to weather extremes (15, 49–51). However, there were varied findings from these studies about the presence of mortality displacement. Mortality displacement has not been found due to the immediate impact of weather extremes in the United States (15, 51). Our finding of death displacement implies that temperature might serve as an indicator for predicting mortality displacement in Vietnam but may need further investigation (23, 52).

The study found that there was a statistically significant relationship between seasonality and mortality among tzhe older people of Hanoi, Vietnam. We also found significant cold-related mortality among these older persons. This finding is consistent with previous studies where the cold season was associated with a high number of deaths among older people (23, 36, 53, 54). The result is similar to a study in the United States that found that overall death rates are higher in winter than in summer (13). A similar relationship of cold-related mortality among older people was observed in Nairobi, though it was not statistically significant (2). Cold temperature has been reported in previous studies to contribute to cardiovascular-related mortality (18, 20, 23, 24, 55). It has been shown that younger people and people with hyperglycemia could enhance susceptibility to cold temperature, whereas old age limits this ability (1, 8). This is true in our study when circulatory disease is the leading cause of death under the study period (23.5% of total death) among older people. Our findings suggest that temperature-related mortality in the cold seasons is a significant public health issue in countries with tropical climates like Vietnam. The finding may have implications for developing intervention strategies to reduce temperature-related impacts. Such strategies could include improving housing conditions and providing insulation or heating facilities during the cold season. A study of cause-specific mortality and temperature relationships among the older people in Vietnam is necessary for understanding the relationship.

However, some studies have also found heat-related mortality (3, 4, 26, 49, 50, 56), and this difference might be explained by differences in time scale for the temperature–mortality data analyzed. Previous studies used the daily data to measure the association between temperature and mortality, but in our study, we used monthly aggregated data that might obscure the relationship between temperature and mortality in heat waves. This suggests that future studies should seek to use daily data in studying refined temperature–mortality relationships.

Our study also found that females were at higher risk of death in cold weather than males. Studies in Europe (3) and China (29), including Hong Kong (7, 8), found that females were at greater risk in high temperature, but so far, none of the studies reported the higher risk of females during cold seasons. Nevertheless, the existing evidence seems to indicate that weather-related impacts are more pronounced among females compared to males. The lower capability of producing maximum heat vasoconstriction puts females at greater risk during cold spells (57–59). The result suggests that the cold–mortality relationship among females needs further investigation to establish possible factors explaining the association.

## Conclusions

This study establishes the temperature–mortality relationship and quantifies seasonality in mortality among older people in Hanoi, Vietnam. The winter excess mortality in the study region can be compared to estimates from Central and Southern European populations. Older people are more susceptible to cold weather, and females appear to be at greater risk. Attention should be provided to protect vulnerable subpopulations from daily weather variations. A comfortable temperature in living conditions and increased attention to vulnerable groups during the cold weather extremes are necessary preventive measures.

However, we acknowledge some limitations of our study. Firstly, the study used monthly data for mortality, which might not be the ideal, and therefore we were not able to establish lag effects of temperature. Despite this limitation, the study still provides some evidence toward understanding weather variability health impacts. In addition, other factors are known to be associated with mortality, such as living conditions, family and social support, access to medical care (14), and indicators of socioeconomic status of the city population (53); these were not adjusted for the current study due to lack of information. Finally, our analysis did not include other factors that may influence the temperature–mortality relationship. We acknowledged this limitation because the data on mortality were available in aggregate form. However, we do not expect the results to differ much since we controlled for season and trend in the time-series model. This is because for the time-series model, the results are not affected by not including variables that vary at the time scale used for modeling. For example, including rainfall is not likely to change the results. Overall, the study provides a relevant contribution to the research topic.
